# Quality of web-based information about the coronavirus disease 2019: a rapid systematic review of infodemiology studies published during the first year of the pandemic

**DOI:** 10.1186/s12889-022-14086-9

**Published:** 2022-09-13

**Authors:** Jenny Stern, Susanne Georgsson, Tommy Carlsson

**Affiliations:** 1 Department of Women’s and Children’s Health, Uppsala University, MTC-huset, Dag Hammarskjölds väg 14B, 1 tr, SE-75237 Uppsala, Sweden; 2 Sophiahemmet University, Stockholm, Sweden; 3 Department of Clinical Science, Intervention and Technology, Karolinska Institute, Stockholm, Sweden; 4 The Swedish Red Cross University, Huddinge, Sweden

**Keywords:** Communicable diseases, Consumer health information, COVID-19, Health education, Internet, Communication, SARS-CoV-2

## Abstract

**Background:**

Following the outbreak of the coronavirus disease 2019, adequate public information was of outmost importance. The public used the Web extensively to read information about the pandemic, which placed significant responsibility in, for many, an unfamiliar situation as the disease spread across the globe. The aim of this review was to synthesize the quality of web-based information concerning the coronavirus disease 2019 published during the first year of the pandemic.

**Materials and methods:**

A rapid systematic review was undertaken by searching five electronic databases (CINAHL, Communication & Mass Media Complete, PsycINFO, PubMed, Scopus). Empirical infodemiology reports assessing quality of information were included (*n* = 22). Methodological quality and risk of bias was appraised with tools modified from previous research, while quality assessment scores were synthesized with descriptive statistics. Topics illustrating comprehensiveness were categorized with content analysis.

**Results:**

The included reports assessed text-based content (*n* = 13) and videos (*n* = 9). Most were rated good overall methodological quality (*n* = 17). In total, the reports evaluated 2,654 websites or videos and utilized 46 assessors. The majority of the reports concluded that websites and videos had poor quality (*n* = 20). Collectively, readability levels exceeded the recommended sixth grade level. There were large variations in ranges of the reported mean or median quality scores, with 13 of 15 total sample scores being classified as poor or moderate quality. Four studies reported that ≥ 28% of websites contained inaccurate statements. There were large variations in prevalence for the six categories illustrating comprehensiveness.

**Conclusion:**

The results highlight quality deficits of web-based information about COVID-19 published during the first year of the pandemic, suggesting a high probability that this hindered the general population from being adequately informed when faced with the new and unfamiliar situation. Future research should address the highlighted quality deficits, identify methods that aid citizens in their information retrieval, and identify interventions that aim to improve the quality of information in the online landscape.

**Supplementary Information:**

The online version contains supplementary material available at 10.1186/s12889-022-14086-9.

## Background

The rates of infection in the coronavirus disease 2019 (COVID-19) caused by SARS-CoV-2 escalated rapidly following the outbreak in 2019 [1]. The disease has caused considerable morbidity and mortality during its first year, particularly among those of higher ages and with predisposing conditions [2, 3]. Consequently, this significant threat to public health required rapid implementation of a wide range of preventive measures within the first year of the pandemic, with the purpose to mitigate infectious spread and impact mainly through behavioral changes in the general population [4, 5]. In order to reach high public adherence to recommended preventive measures rapidly implemented during the first year, successful dissemination of high-quality accurate information was necessary [6]. There was a high public demand for information about COVID-19 in the initial period of the pandemic and many members of the general population used the Web to search for information about this topic [7, 8]. Indeed, the Web has a potential to disseminate accessible and tailored information [9], possibly acting as a large and useful source of information during an epidemic or pandemic.

The Internet is an immense platform of information, encompassing vast volumes of health-related content that is constantly growing and most of which is freely accessible for the public. With no standard systematic system in place to ensure what is being published online, the literature acknowledges a substantial risk of encountering information of substandard quality when browsing the Web [9–12]. In order to enhance the knowledge of what information the public encounters when accessing the Web, an increasing amount of researchers utilizes methods to assess its quality [11–13]. One aspect of the field supply-based infodemiology concerns systematic methods of evaluating the information that is published on the Web [14]. Studies in various medical fields have consistently indicated that a large majority of websites have substandard quality [10–12], illustrating a problematic situation since the Internet is heavily used by the general population as a source of health-related information [15]. Epidemics and pandemics involve a particular circumstance, since a large proportion of the general population is tasked with sorting through a considerable flow of online information on their own. This process is challenging and involves a high risk of widespread promulgation of misinformation and conspiracy theories, often referred to as being an infodemic [16]. Recently, the importance of measuring the impact of infodemics during health emergencies and understanding the spread of low-quality information in public health research has been emphasized further [17].

Since the first year of the pandemic, the public health scenario has involved new challenges as well as opportunities, in particular through the emergence of variants of the virus and a widespread introduction of vaccines. Nevertheless, the first year of the pandemic will undoubtedly for many citizens be remembered as a frightening and unfamiliar situation in which they were required to apply health-related information in new and challenging ways. To learn how information dissemination can be improved in future health emergencies involving communicable diseases, researchers, health professionals and other stakeholders need to consider the potential issues that emerged during the first phase of the COVID-19 pandemic. While the importance of disseminating high-quality information for the public during an epidemic or pandemic is unquestionable, little is yet known about the quality of web-based information about COVID-19. Thus, the aim of this rapid review was to synthesize the evidence on the quality of web-based information concerning the coronavirus disease 2019, intended for the general population and published during the first year of the pandemic.

## Methods

### Design

To meet the need for evidence synthesis due to the pandemic outbreak of COVID-19, a rapid systematic review was undertaken. Rapid reviews are more structured than literature or narrative reviews and involve components of a systematic review, but with degrees of simplified or omitted steps with the intention to produce evidence in a timely manner [18].

### Search methods

Five electronic databases were used to search for published original articles: (i) CINAHL, (ii) Communication & Mass Media Complete, (iii) PsycINFO, (iv) PubMed and (v) Scopus. The searches were performed 11 December 2020, one year after the first confirmed outbreak. Relevant search terms were identified via the following database vocabularies for indexation: CINAHL Subject Headings, Medical Subject Headings, and PsycINFO subjects. Additional search terms were inspired from a review investigating criteria for quality evaluation of online health information [19]. When applicable, truncation and Boolean operators were used. Details concerning the searches are presented in Additional File 1.

Reports were included based on the following criteria: (i) observational empirical infodemiology study investigating the quality of web-based information about COVID-19 intended for public audiences, (ii) published in 2019 or 2020, (iii) systematic quality assessments of web-based information, and (iv) published in the English language. Reports were excluded if investigating: (i) social media, (ii) peer support communication, (iii) information intended for non-public audiences (e.g. health professionals or stakeholders), and (iv) exclusively investigating news articles, since the aim was not to evaluate these sources as single items containing information about COVID-19. Abstracts, letters, editorials, comments and single case studies were excluded.

**Fig. 1:**
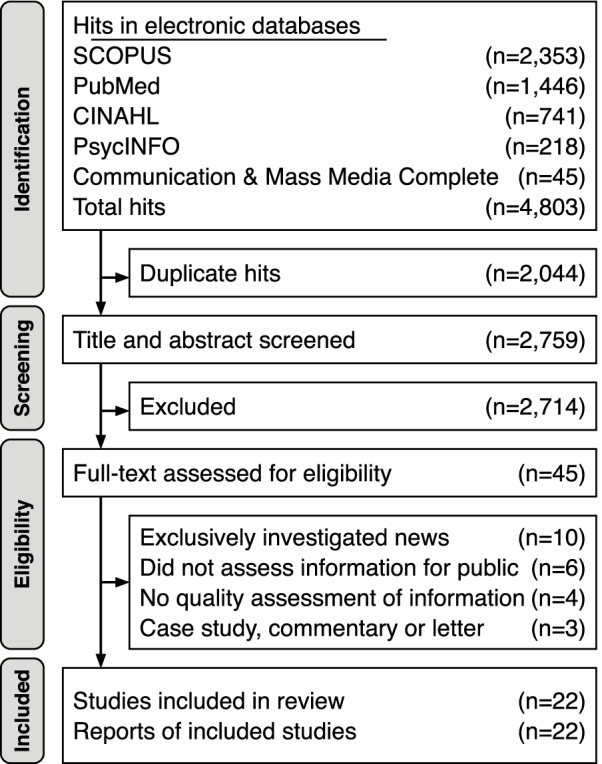
Flowchart of the searches performed in the electronic databases

In total, 4,803 hits were returned from the searches in the databases and 2,044 of these hits were duplicates. After screening the remaining titles and abstracts, 2,714 hits were excluded and thus 45 reports were read in full to assess eligibility. Among these, 23 were excluded after reading the full text document, resulting in 22 reports included in the review. The last author performed the screening and eligibility assessment of titles, abstracts and full-text documents. Figure 1 presents the searches, screening and eligibility assessment. The identified reports were imported to the citation organization software Zotero (version 5.0.96) [20] and the process was managed with the aid of the web-application Rayyan QCRI [21]. No automation tools were utilized during the searches, screening or eligibility assessment of reports.

### Methodological appraisal and risk of bias assessment

The appraisal of the methodological reporting and risk of bias in the included reports was conducted with a pre-specified tool inspired by a previous review of consumer-oriented health information on the Web [10] and the NIH Quality Assessment Tool for Observational Cohort and Cross-Sectional Studies [22], which has been used in a previous review investigating quality of online information [11]. As of yet, no widely established instrument for the systematic assessment of bias in empirical studies investigating quality of websites exists. Therefore, the authors modified the aforementioned instruments to fit the context and inquiry of supply-based infodemiology, and the full appraisal instrument is presented in Additional File 2. The last author performed the quality appraisal and the first author scrutinized the initial appraisal to check for reviewer consistency. Any disagreements were settled through discussion between the two authors until consensus was reached.

### Data extraction and synthesis

The extraction and synthesis was based on the following structure, according to definitions presented in a previous review on quality criteria [19]; (i) readability (‘whether information is presented in a form that is easy to read’), (ii) quality assessments with systematic instruments, (iii) accuracy (’whether a source or information is consistent with agreed-upon scientific findings’), and (iv) comprehensiveness (‘whether a source or information covers a wide range of topics’) or completeness (‘whether necessary or expected aspects of a subject/topic are provided’). The last author developed a data extraction form, inspired by a previous review investigating the quality of online health information for patients and the general population [11]. In regard to quality assessment instruments, total sample and subsample scores were extracted and analyzed with descriptive statistics. Quality assessment scores were determined by calculating the percentage of the total score of the quality assessment instruments (i.e., mean or median score divided by maximum achievable score of the scale/instrument and multiplied by 100). In accordance with previous work [11], the quality assessments were classified as poor (< 44%), moderate (44–80%) and excellent (> 80%). In regard to readability, classification of grade-level scores were based on recommendations from the Joint Commision, stating that patient education materials should be written < 6^th^ grade-level [23], corresponding to > 80 Flesch Reading Ease (FRE) score [24]. In regard to comprehensiveness or completeness, the reported topics covered in the websites were categorized with inductive content analysis by collating the reported topics into categories and subcategories, defined as collections of topics sharing an internally homogeneous and externally heterogeneous content [25]. The reported prevalence of the categorized topics were then extracted and analyzed with descriptive statistics. Lastly, the overall conclusion of each included publication was judged as either (i) poor quality with quality improvements needed, (ii) moderate or varied quality, and (iii) good or excellent quality with no quality improvements needed. RStudio was used to calculate descriptive statistics. The last author performed the data extraction, synthesis and analysis. No assumptions were made in regard to missing or unclear information. When a report only presented quality scores for a certain subset of included websites or videos, this score was considered a total sample score. Scores in studies reporting results from the same samples were omitted in the analysis. The data extracted from the included studies are attached as an Additional File.

## Results

### Methodological appraisal and risk of bias

The median number of adhered methodological quality benchmarks was 5/9 for search process, 2.75/6 for assessment process and 5/7 for the modified NIH assessment tool (Table 1). The overall quality rating of the studies was judged as good (*n* = 17 studies) and fair (*n* = 5 studies). The included studies investigated text-based content (*n* = 13) and videos (*n* = 9), evaluating a total of 2,654 websites and videos (Median = 107.5, Range = 18–321) identified with the search engines Google (*n* = 12 studies), Youtube (*n* = 9 studies), Bing (*n* = 1 study) and Yahoo (*n* = 1 study). Languages assessed in the studies were English (*n* = 17 studies), Spanish (*n* = 6 studies), Mandarin (*n* = 1 study), Korean (*n* = 1 study) and Turkish (*n* = 1 study). Combined, the studies utilized a total of 46 reported assessors (Median = 2, Range = 2–7). In total, assessor qualification was not reported for 33 of the assessors who evaluated the websites or videos, while studies reporting assessor qualification utilized MD with MSc or PhD (*n* = 7 assessors), medical students/trainees (*n* = 3 assessors), assessor with a Master of Science (*n* = 2 assessors), and EdD with MPH (*n* = 2 assessors). Two studies did not report the number of assessors and four exclusively investigated automated readability calculations with no need for quality assessors. Additional File 3 presents the methodological details of the included studies.

**Table 1:** Methodological characteristics of the included reports (*n* = 22)

Benchmarks	Yes, n	No, n	Partially, n	CD/NR, n
Search process^a^				
Search date or period mentioned	19	1	2	-
Search engine or tools explained	21	1	-	-
Justification for engine or tool provided	6	9	7	-
Search terms mentioned	22	-	-	-
Justification for search terms provided	2	13	7	-
Consumer involvement in search process	-	21	1	-
Initial hits in searches reported	7	14	-	1
Language of assessed websites reported	21	-	-	1
IRR for website selection determined	-	20	2	-
Quality assessment process^a^				
Assessors blinded for the source	-	-	-	22
Number of assessors reported	16	-	-	6
Background or qualification of assessors provided	2	10	6	4
Consumer involvement in assessments	-	10	-	12
IRR for assessments determined	12	-	4	6
Criterion standard for measures stated, different from opinion	21	-	1	-
NIH assessment tool for observational/cross-sectional studies^b^				
Research question/objective clearly stated	22	-	-	-
Study population clearly specified and defined	21	1	-	-
Inclusion and exclusion criteria pre-specified, applied uniformly	19	3	-	-
All websites selected from the same or similar populations	21	-	-	1
Sample size justification provided	11	11	-	-
Measures defined, valid, reliable, and implemented consistently	21	-	-	1
Assessors blinded to the source	-	-	-	22

*IRR* Interrater reliability, *CD/NR* Can not determine/not relevant/not reported;

^a^Modified from Eysenbach, et al. [10];

^b^Modified from National Heart, Lung, and Blood Institute[22]

### Results of data extraction and synthesis

#### Conclusions about website quality

The majority of the studies concluded that websites or videos about COVID-19 had poor quality with quality improvements needed (*n* = 19) [26–44]. Three studies concluded that websites or videos had moderate or varied quality [45–47] and none of the included studies concluded that websites or videos had good or excellent quality with no quality improvements needed.

#### Readability

**Fig. 2:**
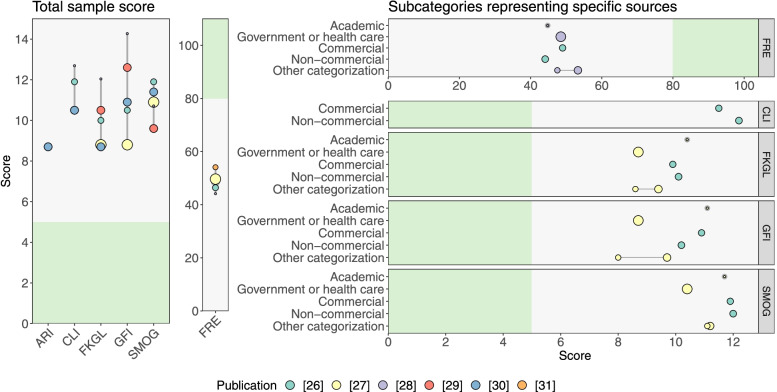
Mean and median readability total sample and subsample scores extracted from the included reports

Readability of written information was evaluated with seven grade-level readability formulas (*n* = 5 reports evaluating *n* = 694 websites) [26–30] and FRE (*n* = 4 reports evaluating *n* = 485 websites) [26–28, 31]. The reported mean or median total sample and subsample scores illustrated a readability exceeding the recommended grade (range = 8.7–14.3) [26–30] and FRE (range = 44.1–54.1) [26–28, 31] levels (Table 2 and Fig. 2). Two reports reported varied prevalence of infographics (7% in one study[30] and 75% in another study [31]). According to one report, the option of viewing similar information in alternative languages was noted in 3% of websites [30].

**Table 2:** The total sample and subsample mean or median quality scores for the included reports (some only reported total sample or subsample score)

**Instrument**	**Reported total sample scores**		**Reported subsample scores**	
	**Studies, n (total included websites, n)**	**Range of reported mean/median**	**Studies, n (total included websites, n)**	**Range of reported mean/median**
Readability				
FKGL	6 (712)	8.7—12.0	2 (340)	8.6—10.4
GFI	6 (712)	8.8—14.3	2 (340)	8.0—11.1
SMOG	6 (712)	9.6—13.4	2 (340)	10.4—12.0
CLI	4 (327)	10.5—12.8	1 (100)	11.5—12.2
ARI	1 (148)	8.7	-	-
FORCAST	1 (18)	11.4	-	-
FRE	4 (485)	44.1—54.1	2 (340)	44.1—53.3
Assessment^a^				
DISCERN^b^	5 (718)	36—80	7 (870)	0—100
JAMA	3 (497)	32—75	3 (495)	25—100
MICI	1 (66)	20	3 (295)	20—28
PEMAT-A^c^	1 (145)	83	-	-
PEMAT-U^c^	1 (145)	41	-	-
EQIP	1 (321)	49	1 (321)	39—67
LIDA	1 (84)	80	-	-
GQS	1 (66)	60	1 (105)	40—80
CSS	-	-	1 (69)	22—70
TCCI	1 (105)	100	1 (105)	60—100

^a^ Mean or median scores presented as percent of maximum achievable score;

^b^ Complete or modified version;

^c^ PEMAT-P subscales reported separately in the report

#### Quality assessments with systematic instruments

**Fig. 3:**
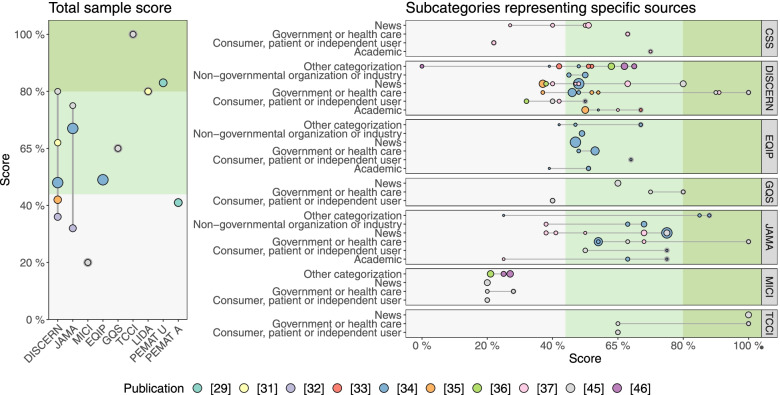
Mean and median quality total sample and subsample scores extracted from the included reports

Ten reports assessed quality using a total of nine instruments (Table 2) [29, 31–37, 45, 46]. The mean or median total sample scores ranged from 20–100% (Table 2), with 13 out of 15 reported scores classified as poor or moderate quality (poor quality: *n* = 4 reports evaluating *n* = 458 websites with *n* = 4 instruments [29, 32, 35, 45]; moderate quality: *n* = 3 reports evaluating *n* = 471 websites with *n* = 5 instruments [31, 34, 45]; excellent quality: *n* = 2 reports evaluating *n* = 211 websites with *n* = 2 instruments [29, 45]) (Fig. 3). Mean or median subsample scores ranged between 0–100% (Table 2), with most scores classified as poor or moderate quality (Fig. 3). Two reports investigated the Health on the Net Foundation Code of Conduct certification and found that ≤ 18% of websites were accredited [31, 32].

The utilized instruments in the reports evaluated the following quality criteria: actionability, authorship, attribution, content (including the COVID-19 specific content prevalence, transmission, signs/symptoms, screening/testing, and treatment/outcome), currency, disclosure, ease of use, flow of information, identification, reliability, sensationalist style, structure, understandability, usability, usefulness, and quality of information about treatment options (Additional File 4).

#### Accuracy assessments

Six reports assessed accuracy by comparing information against current scientific literature or guidelines from health agencies [32, 37, 43, 45–47]. Four reports presented that ≥ 28% of websites contained inaccurate statements (range 58%-28%) [32, 37, 43, 45], while two presented that < 10% of websites contained inaccuracies [46, 47]. One report presented that 11% of the investigated videos included information categorized as hoaxes [40]. Sources with high prevalence or likelihood of inaccurate statements were published by independent users or consumers [45–47] and news [37], while sources with low prevalence or likelihood of inaccurate statements were published by government [37, 45], health care [37] and news [47].

#### Comprehensiveness and completeness assessments

**Fig. 4:**
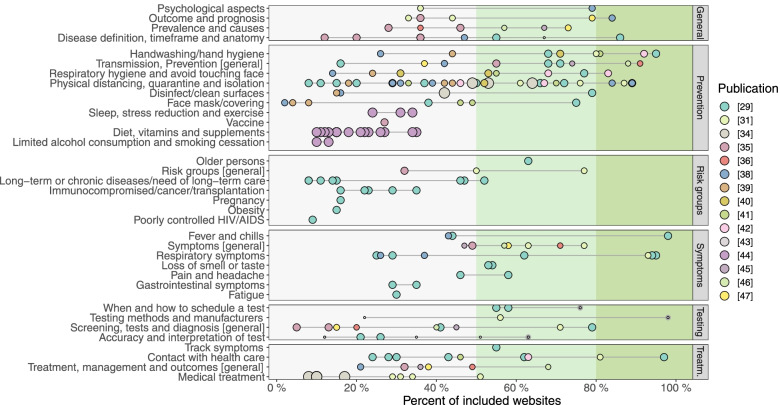
Categories and subcategories extracted from the included reports investigating comprehensiveness

There were large variations in the reported prevalence in all of the six identified categories: general information (range = 12–86%) [29, 31, 35, 36, 38, 43, 45–47], prevention (range = 2–95%) [29, 31, 34–36, 38–47], risk groups (range = 8–77%) [29, 31, 35], symptoms (range = 25–98%) [29, 31, 35, 36, 38, 45–47], testing (range = 5–98%) [29, 31, 35, 36, 43, 45–47] and treatment (range = 8–97%) [29, 31, 34–36, 38, 41, 42, 45–47] (Fig. 4). Additional File 5 shows a detailed presentation on the content and prevalence of each identified category and subcategory.

## Discussion

In this rapid systematic review, 22 reports investigating web-based information during the first year of the COVID-19 pandemic were summarized and synthesized. The methodological appraisal and risk of bias assessment revealed fair to good standards of reporting. The majority of included reports concluded poor quality with quality improvements needed.

In line with previous reviews investigating readability of online health information [12, 48], readability was uniformly determined as exceeding the recommended levels. Health literacy is an essential concept when discussing dissemination of readable information, defined as the ability to access, process and interpret information needed to reach an informed health-related decision [49]. A significant proportion of the global population shows only the lowest or basic levels of literacy [50]. Low health literacy is strongly associated with increased hospitalization and mortality [51], and low coronavirus-related eHealth literacy is associated with less adherence to preventive measures that may mitigate infectious spread [52]. For web-based information to be understood and applied by the general population, it needs to be readable by a large proportion of the population. To a significant extent, the included studies relied on automated readability formulas to draw conclusions. Automated readability formulas has been criticized for being heavily reliant on word and sentence factors, while ignoring other readability-related aspects such as the inclusion of graphics and comprehension [53]. While most studies used automated readability formulas, one study also suggested low prevalence of infographics and few websites containing information in alternative languages. Taken together, our results suggest a problematic situation with most websites exceeding the recommended readability levels and not meeting the literacy found in the diverse population. A large majority of the included studies assessed websites in the English language. During a pandemic, high-quality information needs to reach diverse populations. There is a need to evaluate readability in more languages and by utilizing additional readability assessment methods other than exclusively relying on automated readability formulas, including empirical studies asking the intended end-users to rate the readability of sources.

A range of instruments for quality assessment was utilized in the included reports, which combined illustrate varied quality from the perspectives of several quality criteria, with a tendency towards poor or moderate quality. Our results are highly similar to the findings reported in a previous review investigating the quality of online health information in general [11], indicating that the problematic situation of low quality also is applicable in the context of COVID-19. Website quality is a multidimensional concept involving several different quality criteria [19], of which many were represented in the included studies. In addition to investigating readability, accuracy, and comprehensiveness/completeness, nine quality assessment tools were utilized in the included reports. The assessment tools focus on various aspects of quality (Additional File 4), involving aspects such as usefulness, reliability, content, identification, structure, usability, understandability, and specific information related to COVID-19. There are a number of diverse quality criteria and standards not addressed in the included studies, and thus, it is probable that more studies are needed to fully cover the multidimensional nature of the concept. Nevertheless, the results illustrate that quality of web-based sources about COVID-19 is substandard, based on several criteria and from the perspectives of multiple assessors. Inspecting the reported subsample scores, we did not clearly identify any specific sources that stood out as having particularly low quality, with high variability regardless of source. This calls attention to the likely situation that substandard quality is widely represented within the online landscape, regardless of the type of source behind web-based information. Laypersons who search for health-related information on the Web report low self-efficacy in their ability to successfully identify high-quality online information [48], calling attention to the importance of adequate support needed to encourage the use of adequate information sources. We urge developers, decision-makers and stakeholders to take actions with the aim to increase the probability that the general population encounters high-quality information when accessing the Web to read about COVID-19 or other communicable diseases causing epidemics and pandemics.

There are methodological limitations of this rapid review that needs to be considered when interpreting the results. First, the last author was responsible for screening the hits retrieved in five electronic databases. It is possible that some studies relevant for this review were unintentionally excluded, due to using only one researcher for the screening and eligibility assessment. On the other hand, the use of several databases, citation manager, and screening software implicate a reduced risk of this potential error. Further, we did not screen any grey literature, which may involve a risk of overlooking relevant research not published in the utilized electronic databases for scholarly journals. Second, the last author performed the methodological appraisal and risk of bias assessment, which was scrutinized by the first author until consensus was reached. Due to the lack of widely established instruments for systematic appraisal, the process was conducted by utilizing modified versions of instruments used in previous research [10, 22]. We acknowledge this limitation and encourage the development of valid instruments used for systematic appraisal of methodology and risk of bias in empirical studies investigating quality of web-based information. Third, the quality assessment instruments used in the included studies may involve methodological concerns such as assessor bias and automated readability formulas. The range of methodologies used in the included studies illustrate the multidimensional aspects of the concept of quality criteria, but also calls attention to the need for establishing standards for researchers conducting empirical systematic quality assessments. We appraised the studies as having an overall good methodological quality. However, a number of the investigated quality benchmarks were not reported in the studies, particularly using consumer involvement in the search or assessment process and determining interrater reliability in website selection. Finally, this review highlights reports detailing the quality of web-based information during the first year of the pandemic. The global scenario surrounding COVID-19 is constantly changing and new public health interventions including vaccines have been implemented since the time of our searches. Readers must take time-sensitive aspects into consideration when interpreting our findings. We acknowledge a need for future updated reviews that summarize and synthesize the evidence of public information in later stages of the pandemic. These methodological aspects should be considered and reported when planning future infodemiology studies assessing quality of web-based information.

## Conclusion

This rapid review highlights quality deficits of web-based information about COVID-19 published during the first year of the pandemic, suggesting a high probability that this hindered the general population from being adequately informed. The results call attention to the need of ensuring the dissemination of high-quality information when communicable diseases cause epidemics or pandemics. Considering the high risk of encountering substandard quality when searching for information, developers, decision-makers and stakeholders need to take actions aimed to increase the likelihood of successful dissemination of trustworthy and accurate information that promotes behavioral changes needed to mitigate the spread and impact of epidemics and pandemics. Future research should address the highlighted quality deficits, identify methods that aid citizens in their retrieval of high-quality information during pandemics, and finally, identify interventions that can help change and improve the online landscape.

## Supplementary Information

**Additional file 1.** Search details.

**Additional file 2.** Instruments for methodological assessment.

**Additional file 3.** Methodological presentation and conclusions of the included studies.

**Additional file 4.** Content in the quality assessment instruments used in the included studies.

**Additional file 5.** Topics in the identified subcategories representing completeness/comprehensiveness, with studies reporting each of the topics and ranges of prevalence reported in the publications.

**Additional file 6.** Extracted mean/median scores and prevalence in the included studies.

## Data Availability

All data generated or analysed during this study are included in this published article [and its supplementary information files].

## Abbreviations

ARI: Automated Readability Index
CLI: Coleman-Liau Index
COVID-19: Coronavirus Disease 2019
CSS: Novel COVID-19 Specific Score
EQIP: Ensuring Quality Information for Patients
FKGL: Flesch Kincaid Grade Level
FORCAST: Ford, Caylor, Sticht Formula
FRE: Flesch Reading Ease
GFI: Gunning Fog Index
GQS: Global Quality Score
JAMA: Journal of Americal Medical Association benchmarks
IRR: Interrater Reliability
MICI: Medical Information and Content Index
PEMAT-A: Patient Education Materials Assessment Tool – Actionability subscale
PEMAT-U: Patient Education Materials Assessment Tool – Understandability subscale
SMOG: Simple Measure of Gobbledygook
TCCI: Title–Content Consistency Index
