# Supplementary material for: Quality of web-based information about the coronavirus disease 2019: a rapid systematic review of infodemiology studies published during the first year of the pandemic
Source: BMC Public Health. 2022 Sep 13;22:1734. doi: 10.1186/s12889-022-14086-9 (PMC9467667; doi:10.1186/s12889-022-14086-9)
Supplement: Supplementary file 1 — Additional file 1. Search details. [file 12889_2022_14086_MOESM1_ESM.pdf]

Additional File 1. Search details.

All searches were performed in December 2020

Database: PubMed
Limits: published 2019-2020
Hits: 384
Complete search string: (("Information Dissemination"[Mesh]) OR ("Consumer Health Information"[Mesh]) OR ("Access to Information"[Mesh]) OR ("Information Literacy"[Mesh]) OR (Internet[Mesh]) OR (Web) OR (Websit*)) AND (("Communicable Diseases"[Mesh]) OR (COVID-19[Mesh]) OR (SARS-CoV-2[Mesh])) AND ((”Preventive Health Services”[Mesh]) OR ("Prevention and Control"[Subheading]) OR (”Handwash*”) OR ("Hand Disinfection"[Mesh]) OR ("Hand Hygiene"[Mesh]) OR ("Hygiene"[Mesh]) OR (”Social Distancing”) OR ("Physical Distancing"[Mesh]) OR ("Protective Devices"[Mesh]) OR ("Prevent*"))

Database: PubMed
Limits: published 2019-2020
Hits: 1,062
Complete search string: ((Internet[Mesh]) OR (Web) OR (Websit*) OR (Web-based)) AND ((COVID-19[Mesh]) OR (SARS-CoV-2[Mesh])) AND ((Quality) OR (DISCERN) OR (HON) OR (“JAMA Bench*”) OR (LIDA) OR (QUEST) OR (“Global Quality Score”) OR (“Education Material*”) OR (Evaluat*) OR (Content) OR (Trustworth*) OR (Expertise) OR (Objectivity) OR (Transparen*) OR (Popular*) OR (Understandab*) OR (Relevan*) OR (Familiar*) OR (Accessib*) OR (Identification) OR (Believab*) OR (Accura*) OR (Readab*) OR (Curren*) OR (Navigability) OR (Aesthet*) OR (Interact*) OR (Comprehensiv*) OR (Practical*) OR (Complete*) OR (Useful*) OR (Balance*) OR (Anonymity) OR (Security) OR (Learnab*))

Database: CINAHL
Limits: published 2019-2020, English language
Hits: 413
Complete search string: ((”Information Dissemination") OR (MH "Consumer Health Information+") OR (MH "Access to Information+") OR (MH "Information Literacy+") OR (MH "Counseling+") OR (MH "Internet+") OR (MH "World Wide Web+") OR ("Web") OR ("Websit*") OR ("Counsel*")) AND ((MH "Communicable Diseases+") OR (MH "COVID-19") OR (”SARS-CoV-2”)) AND ((MH "Preventive Health Care+") OR ("Prevention and control") OR (MH "Handwashing+") OR ("Hand Hygiene") OR (MH "Hygiene") OR (MH "Social Distancing") OR ("Physical Distancing") OR (MH "Protective Devices+") OR ("Prevent*"))

Database: CINAHL
Limits: published 2019-2020
Hits: 328
Complete search string: ((MH "Internet+") OR (MH "World Wide Web+") OR ("Web") OR ("Websit*") OR (Web-based)) AND ((MH "COVID-19") OR (”SARS-CoV-2”)) AND ((Quality) OR (DISCERN) OR (HON) OR (“JAMA Bench*”) OR (LIDA) OR (QUEST) OR (“Global Quality Score”) OR (“Education Material*”) OR (Evaluat*) OR (Content) OR (Trustworth*) OR (Expertise) OR (Objectivity) OR (Transparen*) OR (Popular*) OR

(Understandab*) OR (Relevan*) OR (Familiar*) OR (Accessib*) OR (Identification) OR (Believab*) OR (Accura*) OR (Readab*) OR (Curren*) OR (Navigability) OR (Aesthet*) OR (Interact*) OR (Comprehensiv*) OR (Practical*) OR (Complete*) OR (Useful*) OR (Balance*) OR (Anonymity) OR (Security) OR (Learnab*))

Database: Communication & Mass Media Complete
Limits: published 2019-2020
Hits: 10
Complete search string: ((”Information Dissemination") OR ("Consumer Health Information") OR ("Access to Information") OR ("Information Literacy") OR ("Counseling") OR ("Internet") OR ("World Wide Web") OR ("Web") OR ("Websit*") OR ("Counsel*")) AND (("Communicable Diseases") OR ("COVID-19") OR (”SARS-CoV-2”)) AND (("Preventive Health Care") OR ("Prevention and control") OR ("Handwashing") OR ("Hand Hygiene") OR ("Hygiene") OR ("Social Distancing") OR ("Physical Distancing") OR ("Protective Devices") OR ("Prevent*"))

Database: Communication & Mass Media Complete
Limits: published 2019-2020
Hits: 35
((Internet) OR ("World Wide Web”) OR ("Web") OR ("Websit*") OR (Web-based)) AND (("COVID-19") OR (”SARS-CoV-2”)) AND ((Quality) OR (DISCERN) OR (HON) OR (“JAMA Bench*”) OR (LIDA) OR (QUEST) OR (“Global Quality Score”) OR (“Education Material*”) OR (Evaluat*) OR (Content) OR (Trustworth*) OR (Expertise) OR (Objectivity) OR (Transparen*) OR (Popular*) OR (Understandab*) OR (Relevan*) OR (Familiar*) OR (Accessib*) OR (Identification) OR (Believab*) OR (Accura*) OR (Readab*) OR (Curren*) OR (Navigability) OR (Aesthet*) OR (Interact*) OR (Comprehensiv*) OR (Practical*) OR (Complete*) OR (Useful*) OR (Balance*) OR (Anonymity) OR (Security) OR (Learnab*))

Database: PhyscINFO
Limits: published 2019-2020
Hits: 147
((DE "Information Dissemination") OR (DE "Health Information") OR (DE "Consumer Education") OR ("Access to Information") OR (DE "Information Literacy") OR (DE "Counseling") OR (DE"Internet") OR ("World Wide Web") OR (DE”Websites”) OR ("Web") OR ("Websit*") OR ("Counsel*")) AND ((DE "Infectious Disorders") OR ("COVID-19") OR (”SARS-CoV-2”) OR (DE "Coronavirus")) AND ((DE "Preventive Health Services") OR ("Prevention and control") OR ("Handwashing") OR ("Hand Hygiene") OR (DE "Hygiene") OR ("Social Distancing") OR (DE "Physical Distancing") OR ("Protective Devices") OR (DE "Personal Protective Equipment") OR ("Prevent*"))

Database: PhyscINFO
Limits: published 2019-2020
Hits: 71
Complete search string: ((DE"Internet") OR ("World Wide Web") OR (DE”Websites”) OR ("Web") OR ("Websit*") OR (Web-based)) AND (("COVID-19") OR (”SARS-CoV-2”) OR (DE "Coronavirus")) AND ((Quality) OR (DISCERN) OR (HON) OR (“JAMA Bench*”) OR (LIDA) OR (QUEST) OR (“Global Quality Score”) OR (“Education Material*”) OR (Evaluat*) OR (Content) OR (Trustworth*) OR (Expertise) OR (Objectivity) OR (Transparen*) OR (Popular*) OR (Understandab*) OR (Relevan*) OR (Familiar*) OR

(Accessib*) OR (Identification) OR (Believab*) OR (Accura*) OR (Readab*) OR (Curren*) OR (Navigability) OR (Aesthet*) OR (Interact*) OR (Comprehensiv*) OR (Practical*) OR (Complete*) OR (Useful*) OR (Balance*) OR (Anonymity) OR (Security) OR (Learnab*))

Database: Scopus
Limits: published 2019-2020, English language, article
Hits: 756
Complete search string: (TITLE-ABS-KEY ("Information Dissemination" OR "Consumer Health Information" OR "Access to Information" OR "Information Literacy" OR "Counseling" OR "Internet" OR "World Wide Web" OR "Web" OR "Websit*" OR "Counsel*") AND TITLE-ABS-KEY ("Communicable Diseases" OR "COVID-19" OR "SARS-CoV-2") AND TITLE-ABS-KEY ("Preventive Health Care" OR "Prevention and control" OR "Handwash*" OR "Hand Hygiene" OR "Hygiene" OR "Social Distancing" OR "Physical Distancing" OR "Protective Devices" OR "Prevent*")) AND DOCTYPE (ar) AND PUBYEAR > 2018 AND (LIMIT-TO (LANGUAGE , "English"))

Database: Scopus
Limits: published 2019-2020, English language, article
Hits: 1,597
(TITLE-ABS-KEY ("Internet" OR "Web" OR "Websit*" OR "Web-based") AND TITLE-ABS-KEY ("COVID-19" OR "SARS-CoV-2" OR “Coronavirus”) AND TITLE-ABS-KEY (“Quality” OR “DISCERN” OR “HON” OR “JAMA Bench*” OR “LIDA” OR “QUEST” OR “Global Quality Score” OR “Education Material*” OR “Evaluat*” OR “Content” OR “Trustworth*” OR “Expertise” OR “Objectivity” OR “Transparen*” OR “Popular*” OR “Understandab*” OR ”Relevan*” OR ”Familiar*” OR ”Accessib*” OR ”Identification” OR ”Believab*” OR ”Accura*” OR ”Readab*” OR ”Curren*” OR ”Navigability” OR ”Aesthet*” OR ”Interact*” OR ”Comprehensiv*” OR ”Practical*” OR ”Complete*” OR ”Useful*” OR ”Balance*” OR ”Anonymity” OR ”Security” OR ”Learnab*”)) AND DOCTYPE (ar) AND PUBYEAR > 2018 AND (LIMIT-TO (LANGUAGE , "English"))
