# Supplementary material for: Quality of web-based information about the coronavirus disease 2019: a rapid systematic review of infodemiology studies published during the first year of the pandemic
Source: BMC Public Health. 2022 Sep 13;22:1734. doi: 10.1186/s12889-022-14086-9 (PMC9467667; doi:10.1186/s12889-022-14086-9)
Supplement: Supplementary file 2 — Additional file 2. Instruments for methodological assessment. [file 12889_2022_14086_MOESM2_ESM.pdf]

Additional File 2. Instruments for methodological assessment.

Criteria adopted from Eysenbach et al. (modified)[1]	Yes	No	Partially	CD/NA/NR
**1. Search quality (identification of websites)**				
Search date or period mentioned[1]	☐	☐	☐	☐
Search engines mentioned[2]	☐	☐	☐	☐
Justification for selection of engine/s provided	☐	☐	☐	☐
Search terms mentioned	☐	☐	☐	☐
Justification for selection of search term/s provided	☐	☐	☐	☐
Consumer involvement during selection process	☐	☐	☐	☐
Initial hits reported[3]	☐	☐	☐	☐
Language of included websites provided	☐	☐	☐	☐
Interrater reliability for site selection provided	☐	☐	☐	☐
**2. Evaluation quality (assessment of websites)**				
Raters blinded for the source of the website	☐	☐	☐	☐
Number of raters are reported in publication	☐	☐	☐	☐
Background/qualifications of raters are reported	☐	☐	☐	☐
Consumer involvement in assessment process	☐	☐	☐	☐
Interrater reliability for evaluation provided	☐	☐	☐	☐
Criterion standard for quality measure stated and different from personal opinion	☐	☐	☐	☐

[1]Categorized as yes if time period mentioned as date, week or month.
[2]Categorized as no if only web browser is mentioned without details about the search engines used
[3]Categorized as yes if initial hits are reported for either each of the searches or total hits of all searches combined

NIH Quality Assessment Tool for Observational Cohort and Cross-Sectional Studies (modified)[2]	Yes	No	CD/NA/NR
Was the research question or objective in this paper clearly stated?	☐	☐	☐
Was the study population (included websites) clearly specified and defined?	☐	☐	☐
Were the inclusion and exclusion criteria defined and consistent with research question/objective?[1]	☐	☐	☐
Were all the websites selected or identified from the same or similar settings (including the same search engines, search terms and time periods)?	☐	☐	☐
Were the quality criteria clearly defined, valid, pre-specified and implemented consistently?	☐	☐	☐
Was a sample size justification provided?	☐	☐	☐
Were the assessors blinded for the source of the website?	☐	☐	☐

[1]Categorized as no if information about number of hits screened is presented but no other information about inclusion criteria

Overall quality rating of methodological reporting of the publication: Good ☐ Fair ☐ Poor ☐

CD/NA/NR: Cannot determine/Not applicable/Not reported

[1] G. Eysenbach, J. Powell, O. Kuss, E.-R. Sa, Empirical studies assessing the quality of health information for consumers on the world wide web: a systematic review, JAMA. 287 (2002) 2691–2700.
[2] National Heart, Lung and Blood Institute, Study Quality Assessment Tools, (n.d.). https://www.nhlbi.nih.gov/health-topics/study-quality-assessment-tools (accessed February 25, 2021).
