# Supplementary material for: Quality of web-based information about the coronavirus disease 2019: a rapid systematic review of infodemiology studies published during the first year of the pandemic
Source: BMC Public Health. 2022 Sep 13;22:1734. doi: 10.1186/s12889-022-14086-9 (PMC9467667; doi:10.1186/s12889-022-14086-9)
Supplement: Supplementary file 3 — Additional file 3. Methodological presentation and conclusions of the included studies. [file 12889_2022_14086_MOESM3_ESM.pdf]

Additional File 3. Methodological presentation and conclusions of the included studies.

Study	Type	Search process (identification of websites/videos) *[Modified from Eysenbach et al. Empirical studies assessing the quality of health information for consumers on the world wide web: a systematic review. JAMA. 2002;287(20):2691-2700]*									
		Search engine	Search date	Languages	Justification for search engine	Search terms mentioned	Justification for search terms	Consumer involvement in search process	Initial hits reported	IRR website selection	Included websites (n)
[1]	Text	Not reported	No	English	No	Yes	No	No	No	No	100
[2]	Video	Youtube	Yes	English<br>Spanish	Partially	Yes	Partially	No	No	No	100
[3]	Video	Youtube	Yes	English<br>Spanish	Partially	Yes	Partially	No	No	No	100
[4]	Text	Google	Yes	English<br>Spanish	No	Yes	Yes	No	Yes	No	110
[5]	Video	Youtube	Yes	English	Partially	Yes	Partially	No	No	No	113
[6]	Text	Google	Yes	English	Yes	Yes	Yes	No	No	No	321
[7]	Text	Google	Yes	English<br>Spanish	No	Yes	No	No	No	No	80
[8]	Text	Google	Yes	Spanish	Yes	Yes	Partially	No	No	No	120
[9]	Video	Youtube	Yes	Spanish	Yes	Yes	No	No	Cannot determine	No	129
[10]	Text	Google<br>Bing<br>Yahoo	Yes	English	Partially	Yes	No	No	Yes	No	84
[11]	Text	Google	Yes	English	No	Yes	No	No	No	No	48
[12]	Text	Google	Partially	English	Partially	Yes	No	No	No	No	148
[13]	Video	Youtube	Yes	English<br>Mandarin	Yes	Yes	No	No	Yes	No	114
[14]	Text	Google	Yes	English	No	Yes	No	No	No	No	145
[15]	Video	Youtube	Yes	English	Yes	Yes	No	No	No	Partially	69
[16]	Video	Youtube	Partially	Korean	Yes	Yes	Partially	No	Yes	No	105
[17]	Text	Google	Yes	English	Partially	Yes	No	No	No	No	227
[18]	Text	Google	Yes	English	No	Yes	No	No	Yes	No	61
[19]	Video	Youtube	Yes	English	No	Yes	Partially	No	No	No	137
[20]	Text	Google	Yes	Not reported	No	Yes	Partially	Partially	Yes	Partially	27
[21]	Text	Google	Yes	English	No	Yes	No	No	No	No	240
[22]	Video	Youtube	Yes	Turkish	Partially	Yes	No	No	Yes	No	76

Study	Evaluation process (quality assessment of websites/videos) *[Modified from Eysenbach et al. Empirical studies assessing the quality of health information for consumers on the world wide web: a systematic review. JAMA. 2002;287(20):2691-2700]* Assessors blinded	Assessors (n)	Assessor qualification	Consumer involvement in assessment process	IRR assessment	Criterion standard for measures stated, different from opinion	Quality criteria evaluated	
[1]	Not relevant	Not relevant	Not relevant	Not relevant	Not relevant	Yes	Readability:	CLI<br>GFI<br>FKGL<br>FRE<br>SMOG
[2]	Not reported	2	1: MS<br>2: EdD MPH	No	Yes	Yes	Completeness:	Coding sheet
[3]	Not reported	2	1: MS<br>2: EdD MPH	No	Partially	Yes	Completeness	Coding sheet
[4]	Not reported	4	Not reported	Not reported	Partially	Yes	Accuracy:<br><br>Quality assessment:	Comparison literature/guidelines<br>DISCERN instrument<br>HON certification<br>JAMA benchmarks
[5]	Not reported	2	Not reported	Not reported	Yes	Yes	Accuracy:<br><br>Completeness:	Comparison literature/guidelines<br>Coding sheet
[6]	Not reported	6	Not reported	No	Yes	Yes	Completeness:<br>Quality assessment:	Coding sheet<br>DISCERN instrument<br>EQIP<br>JAMA benchmarks
[7]	Not reported	2	1: MD PhD<br>2: MD PhD	No	Yes	Yes	Completeness:	Coding sheet
[8]	Not reported	Not reported	Not reported	Not reported	Not reported	Yes	Completeness:	Coding sheet
[9]	Not reported	2	Not reported	Not reported	Yes	Yes	Completeness:	Coding sheet
[10]	Not reported	2	Not reported	Not reported	Partially	Yes	Accuracy:<br><br>Completeness:<br>Quality assessment:<br><br><br><br>Readability:	Comparison literature/guidelines<br>Coding sheet<br>DISCERN instrument<br>HON certification<br>LIDA<br>FRE
[11]	Not reported	4	Not reported	Not reported	Yes	Yes	Quality assessment:	DISCERN instrument
[12]	Not relevant	Not relevant	Not relevant	Not relevant	Not relevant	Yes	Readability:	ARI<br>CLI<br>FKGL<br>GFI<br>SMOG
[13]	Not reported	3	1: MD<br>2: Medical trainee	No	Yes	Yes	Accuracy:<br><br>Quality assessment:	Comparison literature/guidelines<br>DISCERN instrument<br>MICI
[14]	Not reported	Not reported	Not reported	Not reported	Not reported	Yes	Completeness:<br>Quality	Coding sheet<br>PEMAT

							assessment:<br>Readability:	FKGL<br>GFI<br>SMOG
[15]	Not reported	2	Not reported	No	Yes	Yes	Accuracy:<br><br>Quality assessment:	CSS/comparison literature/guidelines<br>CSS<br>DISCERN instrument<br>JAMA benchmarks
[16]	Not reported	2	1: MD PhD<br>2: MD MSc	No	Yes	Yes	Accuracy:<br><br>Quality assessment:	Comparison literature/guidelines<br>DISCERN instrument<br>GQS<br>JAMA benchmarks<br>MICI<br>TCCI
[17]	Not reported	3	Not reported	No	Yes	Partially	Completeness:	Coding sheet and inductive content analysis
[18]	Not relevant	NA	Not relevant	Not relevant	Not relevant	Yes	Readability:	CLI<br>FKGL<br>FRE<br>GFI<br>SMOG
[19]	Not reported	2	1: Medical student<br>2: Medical student	No	Yes	Yes	Completeness:<br>Quality assessment:	Coding sheet<br>DISCERN instrument
[20]	Not reported	7	Not reported	Not reported	Partially	Yes	Accuracy:<br><br>Completeness:	Comparison literature/guidelines<br>Coding sheet
[21]	Not relevant	Not relevant	Not relevant	Not relevant	Not relevant	Yes	Readability:	GFI<br>FKGL<br>FRE<br>SMOG
[22]	Not reported	2	1: MD<br>2: MD	No	Yes	Yes	Completeness:<br>Quality assessment:	MICI<br>DISCERN instrument<br>MICI

Study	Type	Quality assessment [Modified from National Heart, Lung and Blood Institute. Study Quality Assessment Tools. https://www.nhlbi.nih.gov/health-topics/study-quality-assessment-tools]							Conclusion[a]
		Research question/objective clearly stated	Study population clearly specified and defined	Inclusion and exclusion criteria pre-specified, applied uniformly	All websites selected from the same or similar populations	Sample size justification provided	Criteria defined, valid, pre-specified and implemented consistently	Assessors blinded to the source	
[1]	Text	Yes	No	Yes	Not reported	No	Yes	Not relevant	-
[2]	Video	Yes	Yes	Yes	Yes	No	Yes	Not reported	-
[3]	Video	Yes	Yes	Yes	Yes	No	Yes	Not reported	-
[4]	Text	Yes	Yes	Yes	Yes	No	Yes	Not reported	-
[5]	Video	Yes	Yes	Yes	Yes	No	Yes	Not reported	=
[6]	Text	Yes	Yes	Yes	Yes	Yes	Yes	Not reported	-
[7]	Text	Yes	Yes	No	Yes	No	Yes	Not reported	-
[8]	Text	Yes	Yes	No	Yes	Yes	Yes	Not reported	-
[9]	Video	Yes	Yes	Yes	Yes	Yes	Yes	Not reported	-
[10]	Text	Yes	Yes	Yes	Yes	Yes	Yes	Not reported	-
[11]	Text	Yes	Yes	Yes	Yes	Yes	Yes	Not reported	-
[12]	Text	Yes	Yes	Yes	Yes	No	Yes	Not relevant	-
[13]	Video	Yes	Yes	Yes	Yes	Yes	Yes	Not reported	=
[14]	Text	Yes	Yes	Yes	Yes	Yes	Yes	Not reported	-
[15]	Video	Yes	Yes	Yes	Yes	Yes	Yes	Not reported	-
[16]	Video	Yes	Yes	Yes	Yes	No	Yes	Not reported	=
[17]	Text	Yes	Yes	Yes	Yes	No	Not relevant	Not reported	-
[18]	Text	Yes	Yes	Yes	Yes	Yes	Yes	Not relevant	-
[19]	Video	Yes	Yes	Yes	Yes	Yes	Yes	Not reported	-
[20]	Text	Yes	Yes	Yes	Yes	No	Yes	Not reported	-
[21]	Text	Yes	Yes	No	Yes	Yes	Yes	Not relevant	-
[22]	Video	Yes	Yes	Yes	Yes	No	Yes	Not reported	-

[a] *Conclusion presented as: poor quality with quality improvements needed (-); moderate or varied quality (=); good or excellent quality with no quality improvements needed (+)*

References

[1] C.H. Basch, J. Mohlman, G.C. Hillyer, P. Garcia, Public Health Communication in Time of Crisis: Readability of On-Line COVID-19 Information, Disaster Med Public Health Prep. 14 (2020) 635–637. https://doi.org/10.1017/dmp.2020.151.

[2] C.H. Basch, G.C. Hillyer, Z.C. Meleo-Erwin, C. Jaime, J. Mohlman, C.E. Basch, Preventive Behaviors Conveyed on YouTube to Mitigate Transmission of COVID-19: Cross-Sectional Study, JMIR Public Health Surveill. 6 (2020) e18807. https://doi.org/10.2196/18807.

[3] C.E. Basch, C.H. Basch, G.C. Hillyer, C. Jaime, The Role of YouTube and the Entertainment Industry in Saving Lives by Educating and Mobilizing the Public to Adopt Behaviors for Community Mitigation of COVID-19: Successive Sampling Design Study, JMIR Public Health Surveill. 6 (2020) e19145. https://doi.org/10.2196/19145.

[4] J.Y. Cuan-Baltazar, M.J. Muñoz-Perez, C. Robledo-Vega, M.F. Pérez-Zepeda, E. Soto-Vega, Misinformation of COVID-19 on the Internet: Infodemiology Study, JMIR Public Health Surveill. 6 (2020) e18444. https://doi.org/10.2196/18444.

[5] R.S. D'Souza, S. D'Souza, N. Strand, A. Anderson, M.N.P. Vogt, O. Olatoye, YouTube as a source of medical information on the novel coronavirus 2019 disease (COVID-19) pandemic, Glob Public Health. 15 (2020) 935–942. https://doi.org/10.1080/17441692.2020.1761426.

[6] K.S. Fan, S.A. Ghani, N. Machairas, L. Lenti, K.H. Fan, D. Richardson, A. Scott, D.A. Raptis, COVID-19 prevention and treatment information on the internet: a systematic analysis and quality assessment, BMJ Open. 10 (2020) e040487. https://doi.org/10.1136/bmjopen-2020-040487.

[7] I. Hernández-García, T. Giménez-Júlvez, Assessment of Health Information About COVID-19 Prevention on the Internet: Infodemiological Study, JMIR Public Health Surveill. 6 (2020) e18717. https://doi.org/10.2196/18717.

[8] I. Hernández-García, T. Giménez-Júlvez, Information in spanish on the internet about the prevention of COVID-19, International Journal of Environmental Research and Public Health. 17 (2020) 1–11. https://doi.org/10.3390/ijerph17218228.

[9] I. Hernández-García, T. Giménez-Júlvez, Characteristics of youtube videos in spanish on how to prevent COVID-19, International Journal of Environmental Research and Public Health. 17 (2020) 1–10. https://doi.org/10.3390/ijerph17134671.

[10] R. Jayasinghe, S. Ranasinghe, U. Jayarajah, S. Seneviratne, Quality of online information for the general public on COVID-19, Patient Educ Couns. (2020). https://doi.org/10.1016/j.pec.2020.08.001.

[11] A. Joshi, F. Kajal, S.S. Bhuyan, P. Sharma, A. Bhatt, K. Kumar, M. Kaur, A. Arora, Quality of Novel Coronavirus Related Health Information over the Internet: An Evaluation Study, ScientificWorldJournal. 2020 (2020) 1562028. https://doi.org/10.1155/2020/1562028.

[12] S. Khan, A. Asif, A.E. Jaffery, Language in a Time of COVID-19: Literacy Bias Ethnic Minorities Face During COVID-19 from Online Information in the UK, Journal of Racial and Ethnic Health Disparities. (2020). https://doi.org/10.1007/s40615-020-00883-8.

[13] P. Khatri, S.R. Singh, N.K. Belani, Y.L. Yeong, R. Lohan, Y.W. Lim, W.Z. Teo, YouTube as source of information on 2019 novel coronavirus outbreak: a cross sectional study of English and Mandarin content, Travel Med Infect Dis. 35 (2020) 101636. https://doi.org/10.1016/j.tmaid.2020.101636.

[14] J. Kruse, P. Toledo, T.B. Belton, E.J. Testani, C.T. Evans, W.A. Grobman, E.S. Miller, E.M.S. Lange, Readability, content, and quality of COVID-19 patient education materials from academic medical centers in the United States, Am J Infect Control. (2020). https://doi.org/10.1016/j.ajic.2020.11.023.

[15] H.O.-Y. Li, A. Bailey, D. Huynh, J. Chan, YouTube as a source of information on COVID-19: a pandemic of misinformation?, BMJ Glob Health. 5 (2020). https://doi.org/10.1136/bmjgh-2020-002604.

[16] H. Moon, G.H. Lee, Evaluation of Korean-Language COVID-19-Related Medical Information on YouTube: Cross-Sectional Infodemiology Study, J Med Internet Res. 22 (2020) e20775. https://doi.org/10.2196/20775.

[17] C. Rachul, A.R. Marcon, B. Collins, T. Caulfield, COVID-19 and immune boosting' on the internet: A content analysis of Google search results, BMJ Open. 10 (2020). https://doi.org/10.1136/bmjopen-2020-040989.

[18] T. Szmuda, C. Özdemir, S. Ali, A. Singh, M.T. Syed, P. Słoniewski, Readability of online patient education material for the novel coronavirus disease (COVID-19): a cross-sectional health literacy study., Public Health (Elsevier). 185 (2020) 21–25. https://doi.org/10.1016/j.puhe.2020.05.041.

[19] T. Szmuda, M.T. Syed, A. Singh, S. Ali, C. Özdemir, P. Słoniewski, YouTube as a source of patient information for Coronavirus Disease (COVID-19): A content-quality and audience engagement analysis, Reviews in Medical Virology. 30 (2020). https://doi.org/10.1002/rmv.2132.

[20] S. Taylor-Phillips, S. Berhane, A.J. Sitch, K. Freeman, M.J. Price, C. Davenport, J. Geppert, I.M. Harris, O. Osokogu, M. Skrybant, J.J. Deeks, Information given by websites selling home self-sampling COVID-19 tests: an analysis of accuracy and completeness, BMJ Open. 10 (2020) e042453. https://doi.org/10.1136/bmjopen-2020-042453.

[21] A.P. Worrall, M.J. Connolly, A. O'Neill, M. O'Doherty, K.P. Thornton, C. McNally, S.J. McConkey, E. de Barra, Readability of online COVID-19 health information: a comparison between four English speaking countries, BMC Public Health. 20 (2020) 1635. https://doi.org/10.1186/s12889-020-09710-5.

[22] B. Yuksel, K. Cakmak, Healthcare information on YouTube: Pregnancy and COVID-19, Int J Gynaecol Obstet. 150 (2020) 189–193. https://doi.org/10.1002/ijgo.13246.
