# Supplementary material for: Quality of web-based information about the coronavirus disease 2019: a rapid systematic review of infodemiology studies published during the first year of the pandemic
Source: BMC Public Health. 2022 Sep 13;22:1734. doi: 10.1186/s12889-022-14086-9 (PMC9467667; doi:10.1186/s12889-022-14086-9)
Supplement: Supplementary file 4 — Additional file 4. Content in the quality assessment instruments used in the included studies. [file 12889_2022_14086_MOESM4_ESM.pdf]

Additional File 4. Content in the quality assessment instruments used in the included studies.

Instrument	Quality criteria	Benchmarks/items/questions	Score range
Novel COVID-19 Specific Score (CSS) [1]	Usefulness/completeness	1: Was transmission of COVID-19 explained?	0 (No) – 1 (Yes)
		2: Were typical COVID-19 symptoms described?	0 (No) – 1 (Yes)
		3: Were prevention strategies discussed?	0 (No) – 1 (Yes)
		4: Were potential treatments explained?	0 (No) – 1 (Yes)
		5: Was the epidemiology of COVID-19 briefly discussed using statistics?	0 (No) – 1 (Yes)
		Total CSS score	0 – 5
DISCERN (complete) [2–6]	Subscale 1: reliability	1: Are the aims clear?	1 (No) – 5 (Yes)
		2: Does it achieve its aims? (optional)	1 (No) – 5 (Yes)
		3: Is it relevant?	1 (No) – 5 (Yes)
		4: Is it clear what sources of information were used to compile the publication (other than the author or producer)?	1 (No) – 5 (Yes)
		5: Is it clear when the information used or reported in the publication was produced?	1 (No) – 5 (Yes)
		6: Is it balanced and unbiased?	1 (No) – 5 (Yes)
		7: Does it provide details of additional sources of support and information?	1 (No) – 5 (Yes)
		8: Does it refer to areas of uncertainty?	1 (No) – 5 (Yes)
		Total subscale 1	7 – 40
	Subscale 2: quality of information about treatment options	9: Does it describe how each treatment works?	1 (No) – 5 (Yes)
		10: Does it describe the benefits of each treatment?	1 (No) – 5 (Yes)
		11: Does it describe the risks of each treatment?	1 (No) – 5 (Yes)
		12: Does it describe what would happen if no treatment is used?	1 (No) – 5 (Yes)
		13: Does it describe how the treatment choices affect overall quality of life?	1 (No) – 5 (Yes)
		14: Is it clear that there may be more than one possible treatment choice?	1 (No) – 5 (Yes)
		15: Does it provide support for shared decision-making?	1 (No) – 5 (Yes)
		Total subscale 2	7 – 35
	Subscale 3: Overall quality score	16: Based on the answers to all of the above questions, rate the overall quality of the publication as a source of information about treatment choices	1 (Low) – 5 (High)
		Total DISCERN score	15 – 80
DISCERN (modified) [1,7–9]		Are the aims clear and achieved?	0 (No) – 1 (Yes)
		Are reliable sources of information used? (i.e., publication cited, speaker is an epidemiologist or medical professional)	0 (No) – 1 (Yes)
		Is the information presented balanced and unbiased?	0 (No) – 1 (Yes)
		Are additional sources of information listed for patient reference?	0 (No) – 1 (Yes)
		Are areas of uncertainty mentioned?	0 (No) – 1 (Yes)
		Total modified DISCERN score	0 – 5
EQIP [3]	Content (18)	1: Initial definition of which subjects will be covered	0 (No) – 1 (Yes)
		2: Coverage of the above-defined subjects (if "no" above, does not apply)	0 (No) – 1 (Yes)
		3: Description of the medical problem	0 (No) – 1 (Yes)
		4: Definition of the purpose of the medical intervention	0 (No) – 1 (Yes)
		5: Description of treatment alternatives (including no treatment)	0 (No) – 1 (Yes)
		6: Description of the sequence of the medical procedure	0 (No) – 1 (Yes)
		7: Description of qualitative benefits (e.g. improved mobility)	0 (No) – 1 (Yes)
		8: Description of quantitative benefits (e.g. "40% of patients regain hand mobility")	0 (No) – 1 (Yes)

		9: Description of qualitative risks and side-effects	0 (No) – 1 (Yes)
		10: Description of quantitative risks and side-effects (e.g. ‘‘two thirds of patients experience headache’’)	0 (No) – 1 (Yes)
		11: Addressing quality of life issues (may not apply if very short intervention)	0 (No) – 1 (Yes)
		12: Description of how potential complications will be dealt with (e.g. ‘‘if you feel nauseous we will change the medication’’)	0 (No) – 1 (Yes)
		13: Description of precautions that the patient may take (e.g. ‘‘do not eat 6 h before anaesthesia’’)	0 (No) – 1 (Yes)
		14: Mention of alert signs that the patient may detect (e.g. ‘‘if you feel a burning sensation call the nurse’’)	0 (No) – 1 (Yes)
		15: Addressing medical intervention cost and insurance issues	0 (No) – 1 (Yes)
		16: Specific contact details for hospital services	0 (No) – 1 (Yes)
		17: Specific details of other sources of reliable information/support	0 (No) – 1 (Yes)
		18: The document covers all relevant issues on the topic (summary item for all content criteria)	0 (No) – 1 (Yes)
	Identification (6)	19: Date of issue or revision	0 (No) – 1 (Yes)
		20: Logo of the issuing body	0 (No) – 1 (Yes)
		21: Name of persons or entities that produced the document	0 (No) – 1 (Yes)
		22: Name of persons or entities that financed the document	0 (No) – 1 (Yes)
		23: Short bibliography of evidence-based data used in the document	0 (No) – 1 (Yes)
		24: The document states if and how patients were involved/consulted in its production	0 (No) – 1 (Yes)
	Structure (12)	25: Use of everyday language, explains complex words or jargon	0 (No) – 1 (Yes)
		26: Use of generic names for all medications or products	0 (No) – 1 (Yes)
		27: Use of short sentences (<15 words on average)	0 (No) – 1 (Yes)
		28: The document personally addresses the reader	0 (No) – 1 (Yes)
		29: The tone is respectful	0 (No) – 1 (Yes)
		30: Information is clear (no ambiguities or contradictions)	0 (No) – 1 (Yes)
		31: Information is balanced between risks and benefits	0 (No) – 1 (Yes)
		32: Information is presented in a logical order	0 (No) – 1 (Yes)
		33: The design and layout are satisfactory (excluding figures or graphs)	0 (No) – 1 (Yes)
		34: Figures or graphs are clear and relevant (if absent, ‘does not apply’)	0 (No) – 1 (Yes)
		35: The document has a named space for the reader’s notes	0 (No) – 1 (Yes)
		36: The document includes a consent form, contrary to recommendations	0 (No) – 1 (Yes)
		Total EQIP score	0 – 100%
Global Quality Score (GQS) [8]	Flow of information, ease of use and usefulness	1: Poor quality, poor flow of the site, most information missing, and not at all useful for patients<br>2: Generally poor quality and poor flow, some information listed, but many important topics missing, and of very limited use to patients<br>3: Moderate quality, suboptimal flow, some important information is adequately discussed but other is poorly discussed, and somewhat useful for patients<br>4: Good quality and generally good flow, most of the relevant information is listed but some topics not covered, and useful for patients<br>5: Excellent quality, excellent flow, and very useful for patients	1 (Poor quality) – 5 (High quality) [one of the items are chosen]
JAMA benchmarks [1–3,8]	Authorship	Authors and contributors, their affiliations, and relevant credentials should be provided	Yes – No
	Attribution	References and sources for all content should be listed clearly, and all relevant copyright information noted	Yes – No
	Disclosure	Original: Web site "ownership" should be prominently and fully disclosed, as should any sponsorship, advertising, underwriting, commercial funding arrangements or support, or potential conflicts of interest. This includes arrangements in which links to other sites are posted as a result of financial considerations. Similar standards should hold in discussion forums<br>Modified: “Ownership” should be prominently and fully disclosed, as should any sponsorship, advertising, underwriting, commercial funding arrangements or support, or potential conflicts of interest	Yes – No

	Currency	Dates that content was posted and updated should be indicated	Yes – No
		Total score (adhered benchmarks)	1 – 4
LIDA [4]	Usability (Clarity)	1: Is there a clear statement of who this web site is for?	0 (Never) – 3 (Always)
		2: Is the level of detail appropriate to their level of knowledge?	0 (Never) – 3 (Always)
		3: Is the layout of the main block of information clear and readable?	0 (Never) – 3 (Always)
		4: Is the navigation clear and well structured?	0 (Never) – 3 (Always)
		5: Can you always tell your current location in the site?	0 (Never) – 3 (Always)
		6: Is the colour scheme appropriate and engaging?	0 (Never) – 3 (Always)
	Usability (Consistency)	7: Is the same page layout used throughout the site?	0 (Never) – 3 (Always)
		8: Do navigational links have a consistent function?	0 (Never) – 3 (Always)
		9: Is the site structure (categories or organisation of pages) applied consistently?	0 (Never) – 3 (Always)
	Usability (Functionality)	10: Does the site provide an effective search facility?	0 (Never) – 3 (Always)
		11: Does the site provide effective browsing facilities?	0 (Never) – 3 (Always)
		12: Does the design minimise the cognitive overhead of using the site?	0 (Never) – 3 (Always)
		13: Does the site support the normal browser navigational tools?	0 (Never) – 3 (Always)
		14: Can you use the site without third party plug-ins?	0 (Never) – 3 (Always)
	Usability (Engagability)	15: Can the user make an effective judgment of whether the site applies to them?	0 (Never) – 3 (Always)
		16: Is the web site interactive?	0 (Never) – 3 (Always)
		17: Can the user personalise their experience of using the site?	0 (Never) – 3 (Always)
		18: Does the web site integrate non-textual media?	0 (Never) – 3 (Always)
	Reliability (Currency)	19: Does the site respond to recent events?	0 (Never) – 3 (Always)
		20: Can users submit comments on specific content?	0 (Never) – 3 (Always)
		21: Is site content updated at an appropriate interval?	0 (Never) – 3 (Always)
	Reliability (Conflicts of interest)	22: Is it clear who runs the site?	0 (Never) – 3 (Always)
		23: Is it clear who pays for the site?	0 (Never) – 3 (Always)
		24: Is there a declaration of the objectives of the people who run the site?	0 (Never) – 3 (Always)
	Reliability (Content production)	25: Does the site report a clear content production method?	0 (Never) – 3 (Always)
		26: Is this a robust method?	0 (Never) – 3 (Always)
		27: Can the information be checked from original sources?	0 (Never) – 3 (Always)
		Total EQIP score	0 – 100%
Medical information and content index (MICI) [7–9]	Prevalence	1: Number of confirmed cases reported<br>Alternatively: Number of confirmed or suspected cases reported in South Korea [8]<br>2: Number of suspected cases reported<br>Alternatively: Number of survivors or patients who tested positive again after being declared recovered in Korea [8]<br>3: Number of deaths reported<br>4: Number of countries involved<br>5: Number/proportion of patients who are severely ill	0 – 5 (each adhered item given one point and item scores summarized)
	Transmission	1: Location of origin of virus<br>Alternatively: Transmission (including droplet transmission, close contact transmission, ventilation, and regular disinfection) [8]<br>2: Zoonotic transmission (ie: contact with animals)<br>Alternatively: Hygiene (including washing hands and using a mask) [8]<br>3: Human to human Transmission<br>Alternatively: Social distancing [8]<br>4: Incubation period	0 – 5 (each adhered item given one point and item scores summarized)

		Alternatively: Reinforcement of social measures to limit mass-gathering events (including postponement of school opening, closure of religious and sports establishments, and cancellation of shows and festivals) [8]<br>5: Transmission route via droplets (include: precautionary measures of wearing mask , handwashing)<br>Alternatively: Self-isolation [8]	
	Signs and symptoms	1: Fever<br>2: Upper respiratory tract symptoms (cough, sore throat, runny Nose)<br>Alternatively: Respiratory symptoms (including cough, sputum, sore throat, runny nose, and shortness of breath) [8]<br>3: Lower respiratory tract Symptoms (pneumonia)/ shortness of breath<br>Alternatively: Myalgia, fatigue, lethargy, and chest discomfort [8]<br>4: Myalgia, arthralgia, lethargy<br>Alternatively: Loss of smell or taste [8]<br>5: Diarrhea<br>Alternatively: Abdominal discomfort and diarrhea [8]<br>Alternatively: Gastroenteritis-like symptoms [9]	0 – 5 (each adhered item given one point and item scores summarized)
	Screening and testing	1: Mentions there is a test available<br>Alternatively: Mentions test is possible [8]<br>2: Mentions the test uses respiratory secretion to test<br>Alternatively: Mentions the 1,339 call center to get detailed information [8]<br>3: Mentions that PCR can be used for identification<br>Alternatively: Mentions screening centers (including drive-through screening, community health center, and designated public relief hospitals) [8]<br>Alternatively: Mentions that PCR and immunoglobulin tests can be used for identification [9]<br>4: Shows how this test is done<br>Alternatively: Explains how this test is done (including how much the test costs and when the test result will come out) [8]<br>5: Mentions criteria for testing/screening<br>Alternatively: Mentions the guidelines for testing [8]	0 – 5 (each adhered item given one point and item scores summarized)
	Treatment/outcome	1: Mild symptoms can be self-resolving<br>Alternatively: Triage people who test positive according to their severity (self-isolation, living care centers, and hospitals) [8]<br>2: Some patients becomes ill (mentions hospitalization, ICU admission)<br>Alternatively: Factors influencing severity (smoking, hypertension, diabetes mellitus, malignancy, cardiovascular disease, lung disease, and age) [8]<br>3: Can be dangerous, or lead to death<br>Alternatively: Reactivation in cured patients [8]<br>4: Treatment is supportive but HIV drugs are being used in some circumstances<br>Alternatively: Treatment is supportive, but some medications may be used, such as antimalarial and anti-human immunodeficiency virus agents [8]<br>Alternatively: Treatment is supportive and some new treatment options are under consideration [9]<br>5: Vaccination not currently available	0 – 5 (each adhered item given one point and item scores summarized)
		Total MICI score	0-25
PEMAT-P [10]	Understandability	1: The material makes its purpose completely evident	0 (Disagree) – 1 (Agree)
		2: The material does not include information or content that distracts from its purpose	0 (Disagree) – 1 (Agree)
		3: The material uses common, everyday language	0 (Disagree) – 1 (Agree)
		4: Medical terms are used only to familiarize audience with the terms. When used, medical terms are defined	0 (Disagree) – 1 (Agree)
		5: The material uses the active voice	0 (Disagree) – 1 (Agree)

		6: Numbers appearing in the material are clear and easy to understand	0 (Disagree) – 1 (Agree)
		7: The material does not expect the user to perform calculations	0 (Disagree) – 1 (Agree)
		8: The material breaks or "chunks" information into short sections	0 (Disagree) – 1 (Agree)
		9: The material's sections have informative headers	0 (Disagree) – 1 (Agree)
		10: The material presents information in a logical sequence	0 (Disagree) – 1 (Agree)
		11: The material provides a summary	0 (Disagree) – 1 (Agree)
		12: The material uses visual cues (e.g., arrows, boxes, bullets, bold, larger font, highlighting) to draw attention to key points	0 (Disagree) – 1 (Agree)
		13: The material uses visual aids whenever they could make content more easily understood (e.g., illustration of healthy portion size)	0 (Disagree) – 1 (Agree)
		14: The material's visual aids reinforce rather than distract from the content	0 (Disagree) – 1 (Agree)
		15: The material's visual aids have clear titles or captions	0 (Disagree) – 1 (Agree)
		16: The material uses illustrations and photographs that are clear and uncluttered	0 (Disagree) – 1 (Agree)
		17: The material uses simple tables with short and clear row and column headings	0 (Disagree) – 1 (Agree)
		Total PEMAT understandability score	0 – 100%
	Actionability	18: The material clearly identifies at least one action the user can take	0 (Disagree) – 1 (Agree)
		19: The material addresses the user directly when describing actions	0 (Disagree) – 1 (Agree)
		20: The material breaks down any action into manageable, explicit steps	0 (Disagree) – 1 (Agree)
		21: The material provides a tangible tool (e.g., menu planners, checklists) whenever it could help the user take action	0 (Disagree) – 1 (Agree)
		22: The material provides simple instructions or examples of how to perform calculations	0 (Disagree) – 1 (Agree)
		23: The material explains how to use the charts, graphs, tables, or diagrams to take actions	0 (Disagree) – 1 (Agree)
		24: The material uses visual aids whenever they could make it easier to act on the instructions	0 (Disagree) – 1 (Agree)
		Total PEMAT actionability score	0 – 100%
Title–Content Consistency Index (TCCI) [8]	Sensationalist style of a video (gap between title and content)	1: Eye-catching thumbnail pictures and sensationalist headlines to capture attention, but video title does not match the content<br>2: Visually attractive thumbnail, strong emotionally appealing headlines, and only a little relevant information listed<br>3: Some relevant information listed, but large gaps between title and its content<br>4: Most of the relevant information discussed, but small gaps between title and its content<br>5: An excellent title for the content	1 (Poor consistency) – 5 (High consistency) [one of the items are chosen]

*Complete DISCERN was used but questions were categorized in six dimensions [5]

References

[1] H.O.-Y. Li, A. Bailey, D. Huynh, J. Chan, YouTube as a source of information on COVID-19: a pandemic of misinformation?, BMJ Glob Health. 5 (2020). https://doi.org/10.1136/bmjgh-2020-002604.

[2] J.Y. Cuan-Baltazar, M.J. Muñoz-Perez, C. Robledo-Vega, M.F. Pérez-Zepeda, E. Soto-Vega, Misinformation of COVID-19 on the Internet: Infodemiology Study, JMIR Public Health Surveill. 6 (2020) e18444. https://doi.org/10.2196/18444.

[3] K.S. Fan, S.A. Ghani, N. Machairas, L. Lenti, K.H. Fan, D. Richardson, A. Scott, D.A. Raptis, COVID-19 prevention and treatment information on the internet: a systematic analysis and quality assessment, BMJ Open. 10 (2020) e040487. https://doi.org/10.1136/bmjopen-2020-040487.

[4] R. Jayasinghe, S. Ranasinghe, U. Jayarajah, S. Seneviratne, Quality of online information for the general public on COVID-19, Patient Educ Couns. (2020). https://doi.org/10.1016/j.pec.2020.08.001.

[5] A. Joshi, F. Kajal, S.S. Bhuyan, P. Sharma, A. Bhatt, K. Kumar, M. Kaur, A. Arora, Quality of Novel Coronavirus Related Health Information over the Internet: An Evaluation Study, ScientificWorldJournal. 2020 (2020) 1562028. https://doi.org/10.1155/2020/1562028.

[6] T. Szmuda, M.T. Syed, A. Singh, S. Ali, C. Özdemir, P. Słoniewski, YouTube as a source of patient information for Coronavirus Disease (COVID-19): A content-quality and audience engagement analysis, Reviews in Medical Virology. 30 (2020). https://doi.org/10.1002/rmv.2132.

[7] P. Khatri, S.R. Singh, N.K. Belani, Y.L. Yeong, R. Lohan, Y.W. Lim, W.Z. Teo, YouTube as source of information on 2019 novel coronavirus outbreak: a cross sectional study of English and Mandarin content, Travel Med Infect Dis. 35 (2020) 101636. https://doi.org/10.1016/j.tmaid.2020.101636.
[8] H. Moon, G.H. Lee, Evaluation of Korean-Language COVID-19-Related Medical Information on YouTube: Cross-Sectional Infodemiology Study, J Med Internet Res. 22 (2020) e20775. https://doi.org/10.2196/20775.
[9] B. Yuksel, K. Cakmak, Healthcare information on YouTube: Pregnancy and COVID-19, Int J Gynaecol Obstet. 150 (2020) 189–193. https://doi.org/10.1002/ijgo.13246.
[10] J. Kruse, P. Toledo, T.B. Belton, E.J. Testani, C.T. Evans, W.A. Grobman, E.S. Miller, E.M.S. Lange, Readability, content, and quality of COVID-19 patient education materials from academic medical centers in the United States, Am J Infect Control. (2020). https://doi.org/10.1016/j.ajic.2020.11.023.
