# Supplementary material for: Quality of web-based information about the coronavirus disease 2019: a rapid systematic review of infodemiology studies published during the first year of the pandemic
Source: BMC Public Health. 2022 Sep 13;22:1734. doi: 10.1186/s12889-022-14086-9 (PMC9467667; doi:10.1186/s12889-022-14086-9)
Supplement: Supplementary file 5 — Additional file 5. Topics in the identified subcategories representing completeness/comprehensiveness, with studies reporting each of the topics and ranges of prevalence reported in the publications. [file 12889_2022_14086_MOESM5_ESM.pdf]

Additional File 5. Topics in the identified subcategories representing completeness/comprehensiveness, with studies reporting each of the topics and ranges of prevalence reported in the publications.

Category	Subcategory	Topics included in the subcategory (as reported in publication)	Studies (n)	Range (%)
General information	Total category [1–9]		9	12-86
	Disease definition, timeframe and anatomy	Total subcategory [1,5,7,8]	4	12-86
		Anatomy [7]	1	12
		Definition of COVID-19 [5]	1	86
		Incubation period [1,7]	2	20-47
		Positive antibody test does not infer immunity in future infections [8]	1	67
		SARS mention [7]	1	36
		Timeframe of illness [5]	1	55
		Zoonosis nature [7]	1	36
	Outcome and prognosis	Total subcategory [1–3,7]	4	33-84
		Complications [3]	1	33
		Mentions death [1]	1	84
		Outcome/prognosis [2]	1	79
		Prognosis [3,7]	2	36-44
	Prevalence and causes	Total subcategory [2,4,6,7,9]	5	28-73
		Causes [7]	1	28
		Prevalence [2,4,6,9]	4	36-73
		Spread of virus [7]	1	46
	Psychological aspects	Total subcategory [1,3]	2	36-79
		Effects on quality of life [3]	1	36
		Suggest anxiety or fear [1]	1	79
Prevention	Total category [1–15]		15	2-95
	Diet, vitamins and supplements	Total subcategory [15]	1	10-35
		Avoid fat, sugar, processed foods [15]	1	15
		Diet [15]	1	34
		Elderberry [15]	1	11
		Fruits and vegetables [15]	1	26
		Garlic [15]	1	22
		Ginger [15]	1	15
		Herbs and spices [15]	1	21
		Keep hydrated [15]	1	18
		Minerals [15]	1	10
		Probiotics and prebiotics [15]	1	15
		Supplements and vitamins [15]	1	15
		Tea [15]	1	15
		Tumeric [15]	1	13
		Vitamin A [15]	1	12
		Vitamin C [15]	1	35
		Vitamin D [15]	1	23
		Zinc [15]	1	27

	Disinfect/clean surfaces	Total subcategory [1,5,10,11]	4	15-79
		Disinfecting and cleaning surfaces [1,10,11]	3	15-42
		Disinfect high tough surfaces [5]	1	79
	Face mask/covering	Total subcategory [1,5,10,12]	4	2-75
		Description on proper use of masks [5]	1	38
		Face coverings [5]	1	75
		If healthy, wear a mask if you are taking care of a person with suspected COVID-19/use facemask when caring for ill [10,12]	2	8-46
		Use facemask for protecting others when you are ill [1,10]	2	2-4
		Wear a mask if you are coughing or sneezing [12]	1	49
	Hand wash/hygiene	Total subcategory [1,3,5,10,12–14]	7	26-95
		Hand hygiene [1,5,10]	3	26-95
		Hand washing [3,14]	2	71-80
		How to wash hands [5]	1	68
		Wash your hands frequently [12,13]	2	81-92
	Limited alcohol consumption and smoking cessation	Total subcategory [15]	1	10-13
		Limit alcohol [15]	1	13
		Quit smoking [15]	1	10
	Physical distancing, quarantine and isolation	Total subcategory [1,3,5,8,10–14]	9	8-89
		Avoid close contact with ill persons [1,10]	2	18-31
		Define isolation [5]	1	20
		Define social distancing [5]	1	66
		Define quarantine [5]	1	29
		Exposure self-isolate 14 days [5]	1	37
		Isolation [3]	1	76
		Maintain at least 1 meter distance [12,13]	2	67-70
		Maintain social distancing to protect yourself [5]	1	89
		Physical/social distancing [3,11,14]	1	52-87
		Social isolation [11]	1	64
		Quarantine [1,3]	1	61-89
		Restrict travel [1]	1	84
		Separate yourself from other people if you are sick [5]	1	50
		Stay/remain home/indoors [1,10,11]	3	39-49
		If you are sick, stay home/indoors except to get medical care [5]	1	72
		Stay home/indoors if you feel unwell/when ill [1,10,12,13]	4	29-46
		Return after illness when ten days has passed since positive test, if asymptomatic [5]	1	8
		Return after illness when ten days since symptoms first appeared and you are improving [5]	1	11
		Test positive of molecular test should self-isolate [8]	1	39
		Return after illness when three days has passed without fever [5]	1	15
	Respiratory hygiene and avoid touching face	Total subcategory [1,5,10,12–14]	6	14-83
		Avoid touching eyes, nose, and mouth [12–14]	3	31-68
		Cover cough/sneeze with tissue, throw tissue away [1,10]	2	14-24
		Cover your mouth and nose when you cough or sneeze [12,13]	2	68-83

		Respiratory hygiene and avoid touching face [5,14]	2	53-77
	Sleep, stress reduction and exercise	Total subcategory [15]	1	24-34
		Exercise [15]	1	31
		Sleep [15]	1	34
		Stress reduction [15]	1	24
	Transmission, prevention (unspecified subcategory)	Total subcategory [1,2,4–7,9]	7	16-91
		Asymptomatic spread [5]	1	16
		Close contact (<6 feet) [5]	1	71
		Mechanism of transmission [2]	1	37
		Modes of transmission [1]	1	42
		Prevention [7]	1	55
		Respiratory spread [5]	1	68
		Transmission [4]	1	88
		Transmission and precautionary measures [6,9]	2	74-91
	Vaccine	Total subcategory [7]	1	27
Risk groups	Total category [3,5,7]		3	8-77
	Immunocompromized/cancer/ transplantation	Total subcategory [5]	1	16-35
		Cancer patients [5]	1	29
		Chronic immune weakening medications [5]	1	23
		Immune deficiencies [5]	1	22
		Immunocompromized [5]	1	35
		Transplant patients [5]	1	16
	Long-term or chronic diseases/ need of long-term care	Total subcategory [5]	1	8-52
		Asthma [5]	1	14
		Chronic kidney disease [5]	1	15
		Chronic lung disease [5]	1	47
		Diabetes [5]	1	46
		Dialysis [5]	1	8
		Liver disease [5]	1	8
		Long term care facility patients [5]	1	11
		Serious heart conditions [5]	1	52
	Obesity	Total subcategory [5]	1	15
	Persons >65 years	Total subcategory [5]	1	63
	Poorly controlled HIV/AIDS	Total subcategory [5]	1	9
	Pregnancy	Total subcategory [5]	1	16
	Risk groups (unspecified subcategory)	Total subcategory [3,7]	2	32-77
		Advice for high-risk groups [3]	1	50
		At-risk population [7]	1	32
		Risk factors [3]	1	77
Symptoms	Total category [1–7,9]		8	25-98
	Fatigue	Total subcategory [5]	1	50
	Fever and chills	Total subcategory [1,5]	2	43-98
		Chills [5]	1	44
		Fever [1,5]	2	43-98
	Gastrointestinal symptoms	Total subcategory [5]	1	29-35

		Diarrhea [5]	1	35
		Nausea/ vomiting [5]	1	29
	Loss of smell or taste	Total subcategory [5]	1	53-54
		Ageusia (loss of taste) [5]	1	53
		Anosmia (loss of smell) [5]	1	54
	Pain and headache	Total subcategory [5]	1	46-58
		Headache [5]	1	46
		Myalgia [5]	1	58
	Respiratory symptoms	Total subcategory [1,3,5]	3	25-95
		Cough [1,5]	2	37-95
		Nasal congestion [5]	1	25
		Respiratory symptoms (unspecified) [3]	1	93
		Rhinorrhea [5]	1	29
		Shortness of breath [1,5]	2	26-94
		Sore throat [5]	1	62
	Symptoms (unspecified subcategory)	Total subcategory [2–4,6,7,9]	6	47-77
		Alarming symptoms (unspecified) [3]	1	63
		Clinical symptoms (unspecified) [2,4,9]	3	57-71
		Non-respiratory symptoms (unspecified) [3]	1	77
		Signs and symptoms (unspecified) [6]	1	47
		Symptoms (unspecified) [7]	1	49
Testing	Total category [2–9]		8	5-98
	Accuracy and interpretation of test	Total subcategory [5,8]	2	12-63
		Positive and negative predictive value of test [8]	1	12
		Sample size test accuracy is based on [8]	1	12
		Sensitivity of test [8]	1	63
		Specificity of test [8]	1	51
		Test negative of molecular test may still have the disease [8]	1	35
		What does a negative test mean [5]	1	21
		What does a positive test mean [5]	1	26
	Screening, tests and diagnosis (unspecified subcategory)	Total subcategory [2–7,9]	7	5-79
		Antibody test (unspecified) [5]	1	41
		Diagnosis (unspecified)[7]	1	13
		Investigations (unspecified) [3]	1	71
		Radiological findings (unspecified) [7]	1	5
		Screening (unspecified) [4]	1	40
		Screening and tests (unspecified) [2,9]	2	15-20
		Testing (unspecified) [6]	1	45
		Viral test (unspecified) [5]	1	79
	Testing methods and manufacturers	Total subcategory [3,8]	2	22-98
		Explanation whether the test is antibody or molecular [8]	1	98
		Name of manufacturer of test [8]	1	22
		Screening methods [3]	1	56
	When and how to schedule a test	Total subcategory [5,8]	2	55-76
		How to schedule a test [5]	1	55

		When you should take the test [8]	1	76
		Where can a test be obtained [5]	1	58
Treatment	Total category [1–7,9,11–13]		11	8-97
	Contact with health care	Total subcategory [3,5,12,13]	4	24-97
		If you are sick, contact your health care provider [5]	1	97
		Emergency warning signs to seek care immediately [5]	1	62
		Emergency: bluish lips or face [5]	1	24
		Emergency: inability to stay awake [5]	1	28
		Emergency: persistent chest pain [5]	1	30
		Emergency: trouble breathing [5]	1	43
		If you develop fever, cough, and difficulty breathing, seek medical advice [12,13]	2	46-63
		When to seek medical advice [3]	1	81
	Medical treatment	Total subcategory [3,11]	2	8-51
		Antiviral drugs [11]	1	17
		Benefits of treatment [3]	1	34
		Effects of no treatment [3]	1	29
		Fluids (unspecified) [11]	1	10
		Hydroxychloroquine or chloroquine [11]	1	10
		Non- steroidal antiinflammatory drugs [11]	1	8
		Oxygen [11]	1	10
		Risks of treatment [3]	1	31
		Treatment options (unspecified) [3]	1	51
		Ventilation (unspecified) [11]	1	10
	Track symptoms	Total subcategory [5]	1	55
	Treatment, management and outcomes (unspecified subcategory)	Total subcategory [1,2,4,6,7,9]	6	21-68
		Management (unspecified) [7]	1	32
		Treatment (unspecified) [1,2]	2	21-38
		Treatment and outcome (unspecified) [4,6,9]	3	36-68

References

[1] C.H. Basch, G.C. Hillyer, Z.C. Meleo-Erwin, C. Jaime, J. Mohlman, C.E. Basch, Preventive Behaviors Conveyed on YouTube to Mitigate Transmission of COVID-19: Cross-Sectional Study, JMIR Public Health Surveill. 6 (2020) e18807. https://doi.org/10.2196/18807.

[2] R.S. D'Souza, S. D'Souza, N. Strand, A. Anderson, M.N.P. Vogt, O. Olatoye, YouTube as a source of medical information on the novel coronavirus 2019 disease (COVID-19) pandemic, Glob Public Health. 15 (2020) 935–942. https://doi.org/10.1080/17441692.2020.1761426.

[3] R. Jayasinghe, S. Ranasinghe, U. Jayarajah, S. Seneviratne, Quality of online information for the general public on COVID-19, Patient Educ Couns. (2020). https://doi.org/10.1016/j.pec.2020.08.001.

[4] P. Khatri, S.R. Singh, N.K. Belani, Y.L. Yeong, R. Lohan, Y.W. Lim, W.Z. Teo, YouTube as source of information on 2019 novel coronavirus outbreak: a cross sectional study of English and Mandarin content, Travel Med Infect Dis. 35 (2020) 101636. https://doi.org/10.1016/j.tmaid.2020.101636.

[5] J. Kruse, P. Toledo, T.B. Belton, E.J. Testani, C.T. Evans, W.A. Grobman, E.S. Miller, E.M.S. Lange, Readability, content, and quality of COVID-19 patient education materials from academic medical centers in the United States, Am J Infect Control. (2020). https://doi.org/10.1016/j.ajic.2020.11.023.

[6] H. Moon, G.H. Lee, Evaluation of Korean-Language COVID-19-Related Medical Information on YouTube: Cross-Sectional Infodemiology Study, J Med Internet Res. 22 (2020) e20775. https://doi.org/10.2196/20775.

[7] T. Szmuda, M.T. Syed, A. Singh, S. Ali, C. Özdemir, P. Słoniewski, YouTube as a source of patient information for Coronavirus Disease (COVID-19): A content-quality and audience engagement analysis, Reviews in Medical Virology. 30 (2020). https://doi.org/10.1002/rmv.2132.

[8] S. Taylor-Phillips, S. Berhane, A.J. Sitch, K. Freeman, M.J. Price, C. Davenport, J. Geppert, I.M. Harris, O. Osokogu, M. Skrybant, J.J. Deeks, Information given by websites selling home self-sampling COVID-19 tests: an analysis of accuracy and completeness, BMJ Open. 10 (2020) e042453. https://doi.org/10.1136/bmjopen-2020-042453.

[9] B. Yuksel, K. Cakmak, Healthcare information on YouTube: Pregnancy and COVID-19, Int J Gynaecol Obstet. 150 (2020) 189–193. https://doi.org/10.1002/ijgo.13246.

[10] C.E. Basch, C.H. Basch, G.C. Hillyer, C. Jaime, The Role of YouTube and the Entertainment Industry in Saving Lives by Educating and Mobilizing the Public to Adopt Behaviors for Community Mitigation of COVID-19: Successive Sampling Design Study, JMIR Public Health Surveill. 6 (2020) e19145. https://doi.org/10.2196/19145.

[11] K.S. Fan, S.A. Ghani, N. Machairas, L. Lenti, K.H. Fan, D. Richardson, A. Scott, D.A. Raptis, COVID-19 prevention and treatment information on the internet: a systematic analysis and quality assessment, BMJ Open. 10 (2020) e040487. https://doi.org/10.1136/bmjopen-2020-040487.

[12] I. Hernández-García, T. Giménez-Júlvez, Assessment of Health Information About COVID-19 Prevention on the Internet: Infodemiological Study, JMIR Public Health Surveill. 6 (2020) e18717. https://doi.org/10.2196/18717.

[13] I. Hernández-García, T. Giménez-Júlvez, Information in spanish on the internet about the prevention of COVID-19, International Journal of Environmental Research and Public Health. 17 (2020) 1–11. https://doi.org/10.3390/ijerph17218228.

[14] I. Hernández-García, T. Giménez-Júlvez, Characteristics of youtube videos in spanish on how to prevent COVID-19, International Journal of Environmental Research and Public Health. 17 (2020) 1–10. https://doi.org/10.3390/ijerph17134671.

[15] C. Rachul, A.R. Marcon, B. Collins, T. Caulfield, COVID-19 and immune boosting’ on the internet: A content analysis of Google search results, BMJ Open. 10 (2020). https://doi.org/10.1136/bmjopen-2020-040989.
